# Role of Nesfatin-1 in the Reproductive Axis of Male Rat

**DOI:** 10.1038/srep32877

**Published:** 2016-09-07

**Authors:** Xiaoxiao Gao, Kaifa Zhang, Min Song, Xiumei Li, Lei Luo, Yuan Tian, Yunhai Zhang, Yunsheng Li, Xiaorong Zhang, Yinghui Ling, Fugui Fang, Ya Liu

**Affiliations:** 1 Anhui Provincial Laboratory of Animal Genetic Resources Protection and Breeding, College of Animal Sciences and Technology, Anhui Agricultural University, No. 130 of Changjiang West Road, Hefei, Anhui 230036, China; 2 Anhui Provincial Laboratory for Local Livestock and Poultry Genetic Resource Conservation and Bio-Breeding, No. 130 of Changjiang West Road, Hefei, Anhui 230036, China; 3 Department of Animal Veterinary Science, College of Animal Science and Technology, Anhui Agricultural University, No. 130 of Changjiang West Road, Hefei, Anhui 230036, China

## Abstract

Nesfatin-1 is an important molecule in the regulation of reproduction. However, its role in the reproductive axis in male animals remains to be understood. Here, we found that nesfatin-1 was mainly distributed in the arcuate nucleus (ARC), paraventricular nucleus (PVN), periventricular nucleus (PeN), and lateral hypothalamic area (LHA) of the hypothalamus; adenohypophysis and Leydig cells in male rats. Moreover, the concentrations of serum nesfatin-1 and its mRNA in hypothalamo-pituitary-gonadal axis (HPGA) vary with the age of the male rat. After intracerebroventricular injection of nesfatin-1, the hypothalamic genes for gonadotrophin releasing hormone (*GnRH*), kisspeptin (*Kiss-1*), pituitary genes for follicle-stimulate hormone β(*FSHβ*), luteinizing hormone β(*LHβ*), and genes for testicular steroidogenic acute regulatory (*StAR*) expression levels were decreased significantly. Nesfatin-1 significantly increased the expression of genes for 3β-hydroxysteroid dehydrogenase (*3β-HSD*), 17β-hydroxysteroid dehydrogenase (*17β-HSD*), and cytochrome P450 cleavage (*P450scc*) in the testis of pubertal rats, but their levels decreased in adult rats (*P* < 0.05), along with the serum FSH, LH, and testosterone (T) concentrations. After nesfatin-1 addition *in vitro*, T concentrations of the supernatant were significantly higher than that in the control group. These results were suggestive of the role of nesfatin-1 in the regulation of the reproductive axis in male rats.

Nesfatin-1 is an 82-amino acid hormone derived from the nucleobindin 2 (NUCB2) precursor protein^1^. This hormone has the ability to conduct anorectic effects acting at central (hypothalamic) level^2^,^3^,^4^. This hormone is expressed in the regions of the hypothalamus, including the ARC, PVN, and LHA, and it plays a key role in food intake control and metabolism, and previous studies report that the anorectic effects of nesfatin-1 appear to be independent of leptin signaling^1^,^2^,^5^,^6^.

The neuroendocrine regulation of energy balance and reproduction is tightly interlinked. Evidence indicates that nesfatin-1, in concert with hormones that regulate food intake and energy balance, such as growth hormone-releasing hormone (GHRH), resistin, adiponectin and orexin play an important role in the gonad^7^,^8^,^9^,^10^. Feeding and metabolism are mainly regulated by the brain, while reproduction is achieved by the coordinated actions of the HPGA^11^. This axis has a capacity to respond to metabolic cues from energy stores. Therefore, nesfatin-1 might play a role in the central control of reproduction. Preclinical studies have showed that conditions leading to a negative energy balance are known to perturb puberty, for example chronic sub-nutrition or acute fasting and decreased hypothalamic NUCB2 mRNA and protein levels in pubertal females^12^. In addition, hypothalamic NUCB2 expression increased during the pubertal transition, with a significant elevation of its mRNA levels in the LHA, PVN, and the supraoptic nucleus, and a threefold increase of its total protein content^13^. These data suggest that during puberty, the hypothalamic expression of NUCB2 is subjected to precise developmental and metabolic regulation^13^. Recent preliminary data have revealed the involvement of nesfatin-1 signaling in the control of the pituitary gland and gonads. Indeed, nesfatin-1 can stimulate LH secretion in adult male rats and mice^13^. Furthermore, central injections of anti-NUCB2 morpholino oligo nucleotides reduced the circulating LH levels in pubertal female rats^12^. NUCB2 mRNA expression has been detected in the testes of humans, rats, and mice as well as in the rat ovary^12^,^14^. In non-mammalian models, nesfatin-1 administration also resulted in a significant reduction in the serum LH levels at 60 min after administration^11^.

Nesfatin-1-like immunoreactivity was also found in the follicle cells of zebrafish and goldfish ovaries. Incubation of zebrafish follicles with nesfatin-1 significantly reduced the basal germinal vesicle breakdown during oocyte maturation^11^. Previous research into the expression pattern in gastroenteropancreatic tissues, indicated that nesfatin-1 has age- and tissue-specific role in rats during different developmental stages. In the duodenum, NUCB2 mRNA expression levels at postnatal day 27 were higher than the adult. However, the circulating levels of serum nesfatin-1 increase with development^15^. Very little data is available regarding the role of nesfatin-1 in HPGA in male animals during aging. Thus, the present study aimed to gain further insights into the role of nesfatin-1 in the HPGA of male animals. Using male rats as experimental animals, we focused on four specific questions. (1) How is nesfatin-1 distributed in the HPGA? (2) How does the expression of *NUCB2* mRNA change in the HPGA during aging? (3) What are the roles of nesfatin-1 in mediating reproductive hormones and genes of the gonadal axis in pubertal and adult rats *in vivo*? (4) What are the roles of nesfatin-1 in the regulation of T and related genes in Leydig cells *in vitro*? The results of the present study will help us better understand the role of nesfatin-1 in reproductive physiology.

## Results

### Nesfatin-1 distribution in the hypothalamus, pituitary, and testis

**Figure 1:**
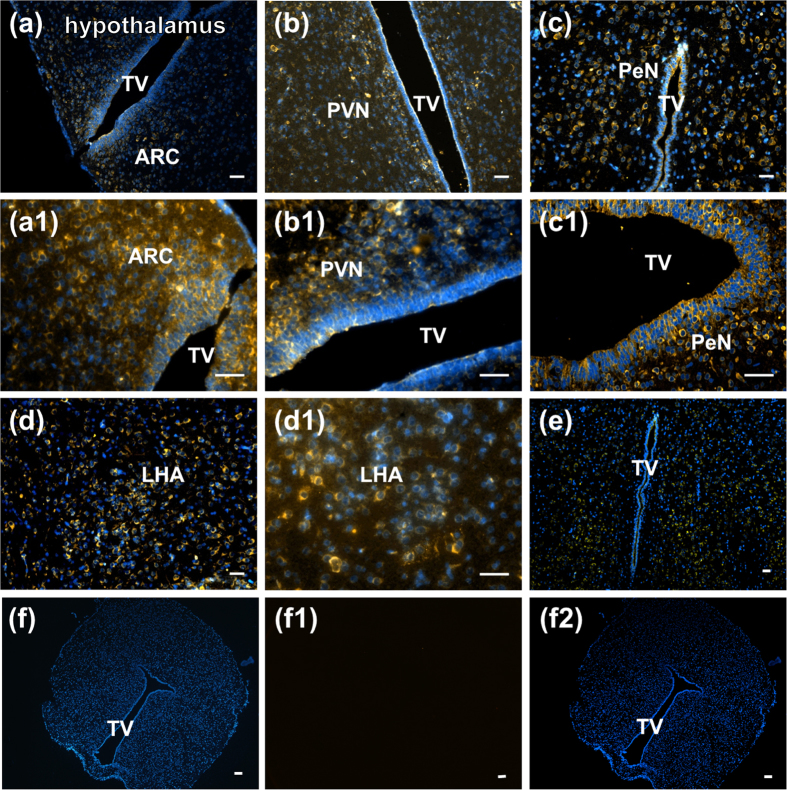
Localization of nesfatin-1-immunopositive cells in hypothalamic nuclei (**a–e**) in male rats. (**a**) and (a1), arcuate nucleus (ARC); (**b**) and (b1), paraventricular nucleus (PVN); (**c**) and (c1), periventricular nucleus (PeN); (**d**) and (d1), lateral hypothalamic area (LHA); (**e**), whole hypothalamic area; (**f**), (f1,f2), negative control. TV: third ventricle, bar = 100 μm.

**Figure 2:**
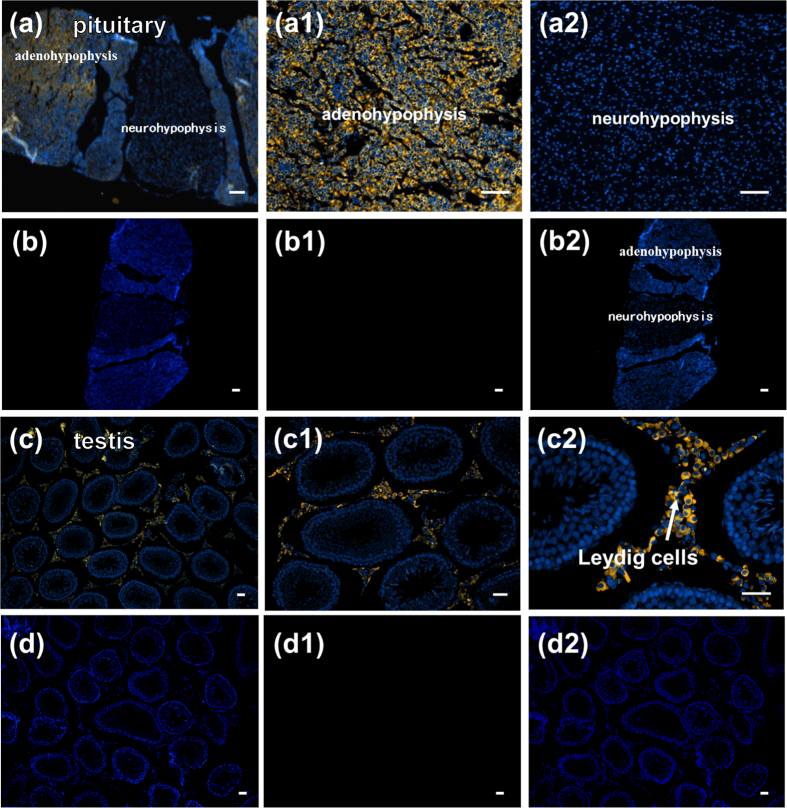
Localization of nesfatin-1-immunopositive cells in pituitary gland (**a**), (a1,a2) and testis (**c**), (c1,c2). (**b**), (b1,b2), negative control in pituitary gland; (**d**), (d1,d2), negative control in testis. bar = 100 μm.

Nesfatin-1 immunoreactivity (IR) was detected in the perikarya of brain cells in the ARC Fig. 1(a), PVN Fig. 1(b), PeN Fig. 1(c), and LHA Fig. 1(d) of the rat hypothalamus, and the adenohypophysis Fig. 2(a,b) and Leydig cells Fig. 2(c,d) of the testis. No IR was found for nesfatin-1 in the neurohypophysis Fig. 2(a) and spermatogenic cells Fig. 2(c), which were stained with the omission of the primary antibodies.

### Serum nesfatin-1 levels and its mRNA in the hypothalamus, pituitary, and testis at different developmental stages

**Figure 3:**
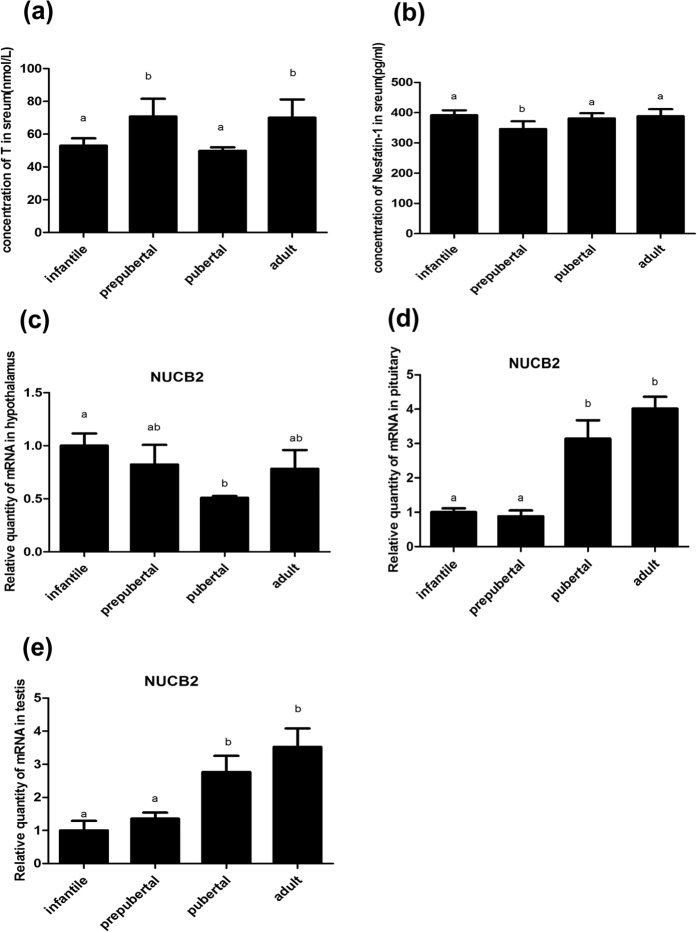
Changes in the serum testosterone (**a**), nesfatin-1 concentrations (**b**) and *NUCB2* mRNA concentrations in the hypothalamus (**c**), pituitary (**d**) and testis (**e**) of infantile (10 d), prepubertal (25 d), pubertal (45 d), and adult (65 d) rats. The values are means ± SEM (n = 5). Means with different letters indicate significant difference (*P* < 0.05).

The serum T levels, nesfatin-1 concentrations and *NUCB* mRNA levels in HPGA were concurrently measured in samples from male rats at different stages of postnatal development. The serum T level was lower (*P* < 0.05) in pubertal (45 d) than prepubertal (25 d) and adult (65 d) rats (Fig. 3(a)). The serum nesfatin-1 concentration was the lowest in prepubertal rats (25 d; *P* < 0.05), and no difference was found between the serum nesfatin-1 levels of prepubertal and adult rats (Fig. 3(b)). RT-PCR revealed that the *NUCB2* mRNA level in the hypothalamus was the highest (*P* < 0.05) in infant (10 d) rats, and this level sharply decreased thereafter, with pubertal rats showing the lowest expression *of NUCB2 (P* < 0.05), and a subsequent increase to moderate level in adult rats Fig. 3(c). No difference was found between the *NUCB2* levels of prepubertal and adult rats (Fig. 3(c)). In the pituitary, the considerably high expression of *NUCB2* mRNA was seen in pubertal and adult rats (*P* < 0.05) while the prepubertal rats presented the low expression (*P* < 0.05; Fig. 3(d)). The *NUCB2* mRNA expression in the testis increased (*P* < 0.05) with age from 10 to 45 d (Fig. 3(e)). There were no differences in the expression levels in the pituitary and testis between pubertal and adult rats (Fig. 3(de)).

### Serum FSH, LH, and T concentrations and GnRH, Kiss1, FSHβ, LHβ, 3β-HSD, 17β-HSD, StAR and P450scc mRNA expression following nesfatin-1 injection in pubertal (45 d) and adult (65 d) rats *in vivo*

**Figure 4:**
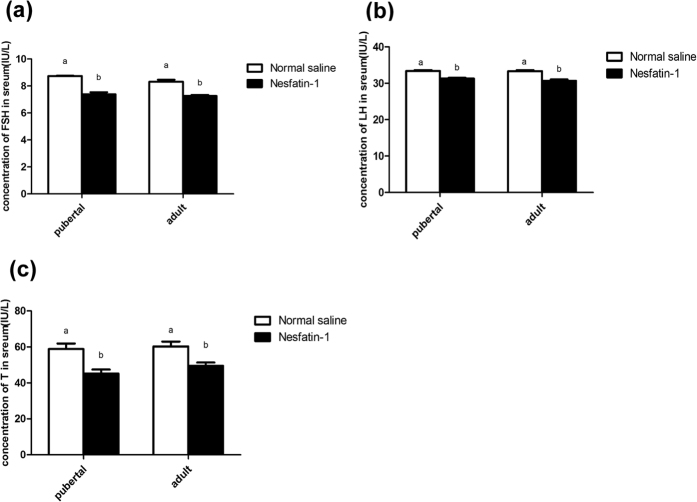
Effect of nesfatin-1 on the levels of serum FSH (**a**), LH (**b**), and T (**c**) levels in pubertal (45 d) and adult (65 d) rats. The values are means ± SEM (n = 5). Means with different letters indicate significant difference (*P* < 0.01).

**Figure 5:**
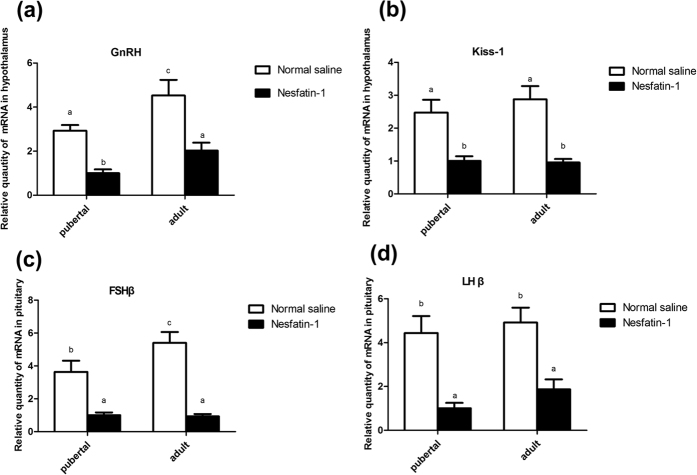
Effect of nesfatin-1 on *GnRH* (**a**) and *Kiss1* (**b**) *mRNA* in the hypothalamus and *FSHβ* (**c**) and *LHβ* (**d**) mRNA in pituitary in pubertal (45 d) and adult (65 d) rats. The values are means ± SEM (n = 5). Means with different letters indicate significant difference (*P* < 0.01).

Intracerebroventricular (icv) injection of nesfatin-1 in pubertal (45 d) and adult (65 d) rats induced a significant decrease in the serum levels of FSH Fig. 4(a), LH Fig. 4(b) and T Fig. 4(c) in both the groups compared with the control (*P* < 0.05). The expressions of *GnRH* Fig. 5(a) and *Kiss1* Fig. 5(b) mRNA in the hypothalamus as well as the *FSHβ* Fig. 5(c) and *LHβ* Fig. 5(d) mRNA in the pituitary were significantly reduced in these groups (*P* < 0.01).

**Figure 6:**
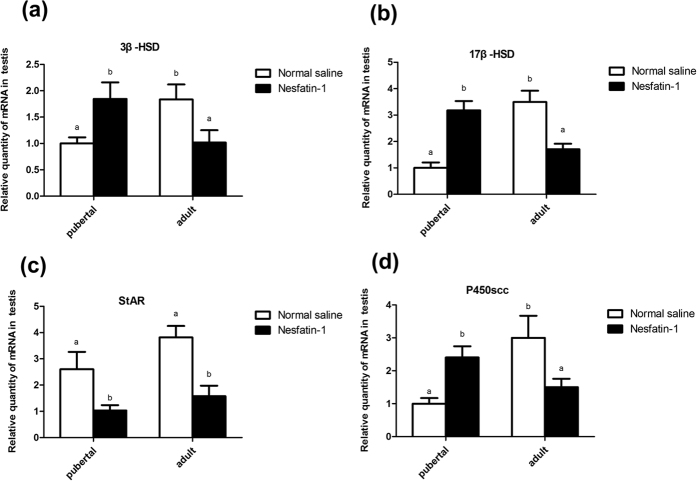
Effect of nesfatin-1 on *3β-HSD* (**a**)*, 17β-HSD* (**b**)*, StAR* (**c**)*, and P450scc* (**d**) mRNA in the testes of pubertal (45 d) and adult (65 d) rats. The values are means ± SEM (n = 5). Means with different letters indicate significant difference (*P* < 0.01).

The *3β-HSD, 17β-HSD, StAR,* and *P450scc* mRNA expression levels in the testes were significantly reduced (*P* < 0.05) after icv injection of nesfatin-1 in adult rats Fig. 6(a–d). However, icv injection of nesfatin-1 in pubertal rats induced a significant increase in the *3β-HSD, 17β-HSD,* and *P450scc* mRNA expression levels in the testes (*P* < 0.05; Fig. 6(a,b,d)). Additionally, the *StAR* mRNA expression level in the testes was significantly decreased in pubertal and adult rats (*P* < 0.05; Fig. 6(c)).

### T concentrations of the supernatant and 3β-HSD, 17β-HSD, StAR, and P450scc mRNA levels in Leydig cells of rats after adding nesfatin-1 *in vitro*

**Figure 7:**
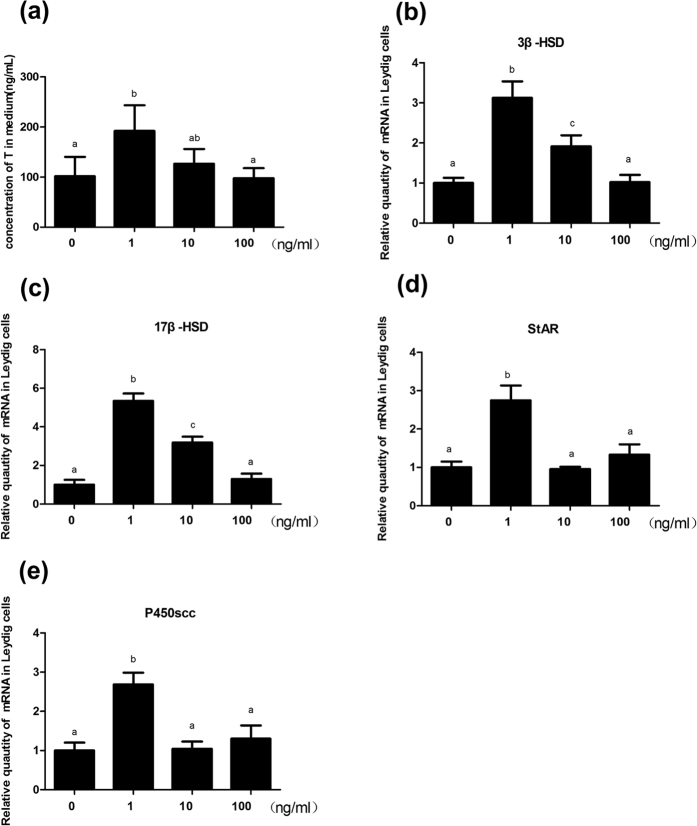
Regulation of testicular T secretion (**a**) and relative levels of *3β-HSD* (**b**), *17β-HSD* (**c**), *StAR* (**d**), and *P450scc* (**e**) mRNA by nesfatin-1 *in vitro*. Leydig cells were incubated with increasing doses of nesfatin-1 (1, 10, and 100 ng/ml), and the concentration of T in the medium was monitored after 48 h of cultivation.

Addition of nesfatin-1 *in vitro* resulted in a significant increase (*P* < 0.05) in the testicular T secretion Fig. 7(a). Addition of 10 and 100 ng/ml of nesfatin-1 had no influence on the testicular T secretion. The *3β-HSD* Fig. 7(b), *17β-HSD* Fig. 7(c), *StAR* Fig. 7(d), and *P450scc* Fig. 7(e) mRNA levels in Leydig cells were significantly higher (*P* < 0.05) than the corresponding levels in the control group after addition of 1 ng/ml of nesfatin-1. When 10 ng/ml of nesfatin-1 was added to rat Leydig cells, there was a significant increase in the expression levels of *3β-HSD* Fig. 7(b) and *17β-HSD* Fig. 7(c) mRNA (*P* < 0.05), but no change in the expressions of *StAR* Fig. 7(d) and *P450scc* Fig. 7(e) mRNA. Furthermore, addition of 100 ng/ml nesfatin-1/NUCB2 had no influence on the expression levels of several genes.

## Discussion

The results of this study revealed the changes in the concentrations of testosterone, nesfatin-1 and *NUCB2* mRNA in the HPGA during different developmental stages, and the resultant changes in the serum T levels. Central administration of nesfatin-1 resulted in differential expression of *GnRH* and *Kiss1* mRNA in the hypothalamus; *FSHβ* and *LH β* mRNA in the pituitary; and *3β-HSD, 17β-HSD, StAR, P450scc,* and *StAR* mRNA in the testis. The T levels of the supernatant and expression levels of *3β-HSD, 17β-HSD, StAR,* and *P450scc* mRNA in the Leydig cells of rats increased after addition of nesfatin-1 *in vitro*.

Nesfatin-1 has been recognized as a hypothalamic satiety factor that, acting in a leptin-independent manner, participates in the central control of appetite^1^,^14^. Indeed, several reports have clarified the role of expression of nesfatin-1 in the stomach and subcutaneous adipose tissue in controlling energy balance throughout development. The results indicated that stomach and the adipose tissue acted on the regulation of circulating nesfatin-1 levels to adapt to different requirements with development^16^,^17^. We found nesfatin-1 IR in cells and altered expression of *NUCB2* mRNA within the HPGA with age, suggesting that nesfatin-1 plays a role in regulating reproduction by modulating the HPGA in male rats. It is well known that in mammals, kisspeptin, a neuropeptide encoded by the *Kiss1* gene, is a potent secretagogue for GnRH^18^,^19^,^20^, and GnRH is a major regulator of LH and FSH synthesis and secretion from the pituitary. In the present study, we found nesfatin-1 IR localized in hypothalamic nuclei especially PVN and ARC, which were implicated in kisspeptin and GnRH secretion, indicating that nesfatin-1 modulates hypothalamic and pituitary hormones. This notion was further strengthened when a single icv injection of nesfatin-1 produced a significant decrease in the serum FSH and LH levels, and the expression of *Kiss1* and *GnRH* mRNAs in the hypothalamus significantly decreased at 60 min after injection. This reduction is suggestive of the inhibitory role of nesfatin-1 on the hypothalamus of male rats. The present results support the previous finding where a single intraperitoneal injection of nesfatin-1 was found to inhibit the expression of *sGnRH* and *cGnRH-II* mRNAs in the forebrain (including the hypothalamus) in fish^11^. However, the mechanisms by which nesfatin-1 regulates *Kiss1* and *GnRH* mRNAs expression remain unclear. Pituitary adenylate cyclase-activating polypeptide (PACAP) directly activates nesfatin-1 neurons in PVN to control feeding and stress response^21^.

In pituitary and testis, the *NUCB2* mRNA expression increased in stages from prepuberty to puberty (*P* < 0.05; Fig. 3(d,e)). However, T level was lower in pubertal than prepubertal rats in our study and other report^22^. So we speculated that the decrease of serum T was involved in nesfatin-1.

We observed a decrease in the *LHβ* and *FSHβ* mRNA expression level in the pituitary of male rats injected with nesfatin-1 via the icv route. The decrease in *LHβ* and *FSHβ* mRNA expression was detected at 60 min after the injection, and these mRNA levels remained significantly lower than those in the control animals. These findings suggest that nesfatin-1 can lower gonadotropin synthesis in the pituitary *in vivo*. As recently reported, progesterone (P4) and 17β-estradiol (E2) could increase the expression of nesfatin-1 in the mouse pituitary gland^23^.We also measured the serum FSH and LH levels in the same rat and found a significant reduction in the circulating FSH and LH levels 60 min after injection. The results supported the previous finding that a single intraperitoneal injection of nesfatin-1 could inhibit the serum FSH and LH levels in female rats^12^. However, some research has clarifyed that nesfatin-1 could stimulate luteinizing hormone (LH) secretion in adult male rats 30 min post injection. We inferred that the different intervals between injection and blood collection may be a reason for the conflicting results. In addition, only pubertal and adult rats were treated by nesfatin-1 in our study; we will explore the nesfatin-1 treatment in prepubertal and infantile rats in further study.

As previously reported, nesfatin-1 was localized in Leydig cells in the testis and Western blot analysis determined a significant increase in the nesfatin-1 levels in the testes of adult rats compared to pubertal rats^14^. However, the mechanisms of nesfatin-1 action in Leydig cells remain unclear^24^. We demonstrated that nesfatin-1 mRNA expression in the testis of rats increased with age from 10 d to 45 d.

T is synthesized in the Leydig cells by a series of enzymes in male animals, such as 3β-HSD, 17β-HSD, StAR, P450scc^25^,^26^,^27^,^28^,^29^.The present study observed a decrease in the 3β-HSD, 17β-HSD, StAR, and P450scc mRNA of the testes in adult rats after icv injection of nesfatin-1.We also found that icv injection of nesfatin-1 in pubertal and adult rats both induced significant decrease in the serum levels of T. However, the icv injection of nesfatin-1 in pubertal (45 d) rats induced a significant increase in the expression levels of *3β-HSD, 17β-HSD,* and *P450scc* mRNA of the testes. We inferred that the differential expression may be associated with the degree of testicular development. Testicles of adult rats are fully mature and pubertal rats are in a transition, and steroid hormone secretion by the gonads is consequently unstable; however, further studies are required to determine the specific mechanism of these genes.

As reported, hCG-stimulated T secretion was enhanced by adding nesfatin-1 to rat testicular explants^14^. We observed significant increases in the T levels secreted from Leydig cells and the *3β-HSD, 17β-HSD, StAR,* and *P450scc* mRNA levels after adding 1 ng/ml nesfatin-1 to the culture medium. However, these findings are the opposite of those obtained following icv injection of nesfatin-1. As previous reported, the production of T from leydig cells would increase as the result of gonadotropin stimulating^30^,^31^. Therfore, We inferred that Leydig cells secrete T would be influenced by the indirect action decreased gonadotropin of after treated by nesfatin-1 *in vivo*. Moreover, the different time of sampling would be involved in production of T, as we test the concentration of T at 1 h and 24 h respectively. The above two factors may be the reasons for the conflicting results.

## Conclusions

The present study demonstrates that: (1) both nesfatin-1 and its mRNA are present in HPGA; (2) concentrations of serum nesfatin-1 and its mRNA in HPGA vary with the age of the rat; (3) nesfatin-1 reduced the serum FSH, LH, and T levels; hypothalamic *GnRH* and *Kiss1* mRNA; pituitary *FSHβ* and *LH β* mRNA; and testis *3β-HSD*,*17β-HSD, StAR,* and *P450scc* mRNA in adult rats; and (4) treatment with 1 ng/ml of nesfatin-1 *in vitro* increased the expression levels of *3β-HSD, 17β-HSD, StAR,* and *P450scc* mRNA in Leydig cells as well as the T levels in the supernatant. Together, these results suggest that nesfatin-1 plays a role in regulating the reproductive axis in male rats.

## Materials and Methods

### Animals

Adult Sprague Dawley rats were purchased from the Experimental Animal Center of Anhui Medical University and allocated into breeding pairs. For the first 19 days after pairing, litters were assessed daily, and the day of birth was considered postnatal day 1. The animals were weaned on day 21. The rats were kept under standard conditions (12:12 h light-dark cycle with lights on between 06:00 and 18:00 h; temperature, 22 ± 1 °C; rat chow and water provided *ad libitum*). The study was approved by the Animal Care and Use Committee of Anhui Agricultural University. The methods were carried out in accordance with the approved guidelines.

### Experimental design

#### Experiment 1. Distribution of nesfatin-1 in the hypothalamus, pituitary, and testis

At puberty (45 d), the rats were deeply anesthetized with 60 mg/kg of 1% pentobarbital sodium. Physiological saline, followed by 4% paraformaldehyde in 0.1 M sodium phosphate buffer (pH 7.4), was perfused through the left ventricle of the heart. After the rats were sacrificed, the hypothalamus, pituitary gland, and testis were removed and fixed in 4% paraformaldehyde for 4 h, and immersed in 30% sucrose in PBS overnight. The sections were sliced to 5-μm thick slices on a freezing microtome (Leica Microsystems, Wetzlar, Germany). The series of sections were collected in antifreeze solution (30% ethylene glycol; 25% glycerol; 0.05 M PBS) and stored at –20 °C until use for immunofluorescence.

#### Experiment 2. Change in the NUCB2 mRNA level in the hypothalamus, pituitary and testis of rats at different developmental stages

The animals were sacrificed at infant (10 d), prepubertal (25 d), pubertal (45 d), and adult (65 d), respectively. After the rats were sacrificed, the hypothalamus, pituitary glands and testes were surgically removed, immediately frozen in liquid nitrogen, and stored at –80 °C until gene analysis.

#### Experiment 3. Analyses of serum follicle-stimulating hormone (FSH), LH, and T levels and expression of GnRH, Kiss1, FSHβ, LHβ, 3β-HSD, 17β-HSD, StAR and P450scc mRNAs following nesfatin-1 treatment

Pubertal (45 d) and adult (65 d) rats were intracerebroventricularly injected with 10 μL nesfatin-1 solution (containing 10 μg nesfatin-1). Animals in the vehicle group (control) received 10 μL saline alone. After 60 min of injection, blood samples were withdrawn, centrifuged at 200 × *g* for 20 min at 4 °C, and the obtained serum was stored at –20 °C until enzyme-linked immunosorbent assay (ELISA) for FSH, LH, and T. The rats were then decapitated, and the hypothalamus, pituitary, and testis tissues were removed and rapidly flash-frozen under liquid nitrogen for total RNA extraction.

#### Experiment 4. Effects of nesfatin-1 on the T level and the expression of 3β-hydroxysteroid dehydrogenase (HSD), 17β-HSD, StAR, and P450scc mRNA expression levels *in vitro*

Rats (two per experiment) were humanely sacrificed by decapitation, and testis samples were collected. Leydig cells were isolated and identified by 3 B-HSD staining as previously described^32^,^33^. Briefly, the cells were plated in 24-well tissue culture plates at a concentration of 1 × 10^5^ cells/well, and were incubated without or with increasing concentrations of nesfatin-1 (10^−9^ to 10^−6^ mol/L) at 37 °C in a humidified atmosphere of 5%CO_2_ and 95% O_2_ for 24 h. The culture medium was then collected to determine the T levels, and Leydig cells were collected for subsequent RNA extraction.

### Fluorescence immunocolocalization of nesfatin-1

Sections were deparaffinized in 100% xylene and rehydrated in a graded ethanol series. Protein Block Serum-Free reagent (Dako) was used for blocking for 10 min. The sections were then incubated in rat anti-nesfatin-1 primary polyclonal antibodies (raised in sheep, 1:500 dilution; AF6895, R&D Systems, USA) for 18 h at room temperature in the hypothalamus. After incubation, the slides were washed thrice with 1 × PBS for 10 min each time at room temperature) and incubated with donkey anti-sheep immunoglobulin (Ig) G-fluorochrome NL557 (1:200 dilution; NL010, R&D Systems, USA) secondary antibody for 1 h at room temperature. The slides were washed thrice with 1 × PBS for 10 min each time at room temperature and mounted with Vectashield mounting medium containing 4, 6-diamidino-2-phenylindole (DAPI; blue nuclear stain; Vector Laboratories). The negative controls were not incubated with primary antibody and were only treated with secondary antibody.

All the images were taken using a fluorescence microscope (Olympus, Japan). Representative images were chosen from a large number of sections taken from all animals. The preabsorption control for the nesfatin-1 primary antibodies used here was validated by immunofluorescence staining with a well-characterized antiserum.

### Total RNA isolation and reverse transcription

Total RNA was extracted using the Trizol reagent (ShineGene, Shanghai, China), according to the manufacturer’s protocol. The concentration of total RNA was quantified at 260 nm with a NanoDrop spectrophotometer (ND-1000, USA). Reverse transcription was performed using EnergicScript^®^ First Strand cDNA Synthesis Kits (ShineGene, Shanghai, China), according to the manufacturer’s protocol^34^. A total of 5 μg of RNA in 20 μL were used for the reverse transcription.

### Real-time PCR

Information on the primers for target genes and housekeeping (*β-actin*) internal control genes are listed in Table 1. The nucleotide sequences for these genes were designed using Primer Express 2.0 software (Applied Biosystems [ABI], Foster City, CA) and were based on NCBI reference sequences for rat. Real-time PCR was performed exactly as described previously^34^. Data were analyzed using FTC2000 Software (Funglyn, Toronto, Canada) and shown as a ratio of analyzed gene expression to *β-actin* and *Ywhaz* (Table 1).

### Determination of nesfatin-1, FSH, LH, and T concentrations

Serum nesfatin-1, FSH, LH, and T concentrations were measured using commercial ELISA kits according to the manufacturer’s instruction (Wuhan Xinqidi Biological Technology, Wuhan, China). Briefly, 100 μL of standard or sample was added to each well and incubated for 2 hours at 37 °C. The liquid was then removed from each well, and 100 μL of anti-biotin antibodies was added, and the wells were incubated for 1 hour at 37 °C. After washing thrice with washing buffer (200 μL) using an autowasher (ELx 50, BIOTEK, USA), 100 μL of horseradish peroxidase (HRP)-conjugated avidin was added to each well and incubated for 1 hour at 37 °C. The plates were washed five times as mentioned above. Then, 90 μL of TMB substrate was added to each well and incubated for 20 minutes at 37 °C. Finally, 50 μL of stop solution was added to each well. Absorbance was determined at 450 nm using a microplate reader (ELx808IU, Biotek, USA). The correlation coefficients were more than 0.95 for the standard curve of the FSH, LH, and T assays, and the intra- and inter-assay coefficients of variation (CVs) were less than 9% and 11%, respectively. The sensitivity and detection range for the FSH assay were 0.5 IU/L and 0.5–10 IU/L, respectively; the corresponding values for the LH assay were 2 ng/L and 2–40 ng/L, respectively; and those for the T assay were 3 nmol/L and 3–160 nmol/L, respectively.

### Statistical analysis

All values are reported as the means ± SEM. Statistical analyses were performed using one-way ANOVA followed by Student’s *t* test. Differences were considered to be significant at *P* < 0.05.

## Additional Information

**How to cite this article**: Gao, X. *et al*. Role of Nesfatin-1 in the Reproductive Axis of Male Rat. *Sci. Rep.* **6**, 32877; doi: 10.1038/srep32877 (2016).

## Figures and Tables

**Table 1:** Real-time PCR primer and size of the amplification products of the target and housekeeping genes.

Gene	Forward primer, 5′-3′	Reverse primer, 5′-3′	Product, bp
*NUCB2*	CCAGACACGGGACTTTATTATG	CCGCTCCTTATCTCCTCTATGT	122
*FSHβ*	AGACCAAACACCCAGAAAG	TCACTATCACACTTGCCACA	140
*GnRH*	GCCGCTGTTGTTCTGTTGAC	CTGGGGTTCTGCCATTTGA	153
*Kiss-1*	TGCTGCTTCTCCTCTGTGTG	CCAGGCATTAACGAGTTCCT	117
*LHβ*	CATAGTCTCCTTTCCTGTGGC	CATTGGTTGAGTCCTGGGA	91
*StAR*	CAACTGGAAGCAACACTCTACA	ACACCTGGCACCACCTTACT	164
*P450scc*	AACGGCACACACAGAATCCAT	AAGAGAGTCGCTGCGTCCTTAG	128
*3β-HSD*	GTGTATGTAGGCAATGTGGC	ACTGGAATCAAGGTGGAGG	180
*17β-HSD*	AGCCAAGAAGTTAGGAGGAAAC	CACAGTTGACAGCCACATCT	125
*β-actin*	CCCATCTATGAGGGTTACGC	TTTAATGTCACGCACGATTTC	150
*Ywhaz*	TTGAGCAGAAGACGGAAGGT	GAAGCATTGGGGATCAAGAA	136
